# Indirect Mitral Annuloplasty Using the Carillon Device

**DOI:** 10.3389/fcvm.2020.576058

**Published:** 2020-11-20

**Authors:** Amar Krishnaswamy, Samir R. Kapadia

**Affiliations:** ^1^Department of Interventional Cardiology, Cleveland Clinic, Cleveland, OH, United States; ^2^Department of Cardiovascular Medicine, Cleveland Clinic, Cleveland, OH, United States

**Keywords:** mitral regurgitation, carillon, percutaneous mitral valve repair, indirect mitral annuloplasty, heart failure

## Abstract

Patients with functional, or secondary, mitral regurgitation (FMR, SMR) often face significant symptoms that lead to functional decline as well as hospitalization and even death. Traditional mitral annuloplasty is an important treatment option for patients with FMR, but surgical risk and durability are important limitations. Percutaneous strategies are therefore a welcome alternative. The Carillon device utilizes the relationship of the coronary sinus and the mitral annulus to effect an “indirect” annuloplasty. Early series' and recent randomized trials suggest echocardiographic and clinical benefit with a relatively straight-forward implantation technique and low rate of significant complications.

## Introduction

Patients with functional, or secondary, mitral regurgitation (FMR, SMR) often face significant symptoms that lead to functional decline as well as hospitalization and even death (1). While guideline-directed medical therapy (GDMT) for heart failure is recommended for all, and often provides some relief, many patients remain symptomatic. Unfortunately, surgical mitral valve (MV) repair or replacement for patients with SMR is a poor option, owing to both significant rates of recurrence of SMR as well as a higher level of operative risk and overall mortality due to competing comorbid conditions (2, 3). As a result, the landmark COAPT trial was both practice- and mindset-changing as an example of a mechanical therapy for SMR that was relatively safe and provided both symptomatic and survival benefit for patients with SMR (4).

It is important to realize, however, that the anatomic basis of valve regurgitation can actually be quite variable among the spectrum of patients with SMR. As such, edge-to-edge leaflet repair with devices such as the MitraClip (Abbott Vascular, Minneapolis, MN) or Pascal (Edwards Lifesciences, Irvine, CA) may either not be feasible or provide adequate reduction in SMR for some patients. There is, therefore, a significant need for alternative devices aimed at repairing or replacing the MV. In this review, we will address one of these, the Carillon (Cardiac Dimensions), which is designed to provide an indirect MV annuloplasty *via* the coronary sinus (CS).

## Anatomic Basis for Coronary Sinus (Indirect) Mitral Annuloplasty

Historically, SMR was referred to as “a problem of the ventricle” with left ventricular (LV) dilation causing apical displacement and tethering of the MV leaflets and/or dilation of the MV annulus (MVA) (5). While pathologic LV dilation is an important part of mitral regurgitation (MR), the contemporary understanding additionally implicates either idiopathic annular dilation without LV dilation or atrial fibrillation/flutter alone as the primary etiology in more than one-third of SMR patients (6).

Given the continuity of the atrial endocardium with the mitral leaflets, enlargement of the atrium results in annular dilation and pulls apart the MV leaflets, thereby reducing the coaptation surface (7). Further, “atrializing” the MV leaflets results in tethering of the papillary muscles and chords to the LV.

Understanding the relationship of the CS to the MVA provides an anatomic basis for indirect annuloplasty. The great cardiac vein and the main posterior lateral vein join at the lateral aspect of the heart to form the CS, which then receives other venous tributaries before emptying into the right atrium (RA) (8). Anatomically, the CS lies in the sulcus between the left atrium (LA) and LV, and in all patients sits on the atrial side of the MVA. By analyzing cardiac CT scans of normal and SMR patients, our group has previously demonstrated that the distance between the CS and MVA increases among patients with FMR, perhaps owing to a “flattening” of the typical saddle shape of the MVA (9). Further, the CS “crosses” the left circumflex (LCx) coronary artery in 80% of patients, though with significant variability in the location of crossing (range 37–123 mm from the CS Os).

Owing to the anatomic relationship of the CS to the MVA, and the contribution of annular dilation to most patients with SMR, it therefore stands to reason that reducing the annular dimension may be achieved by remodeling through the CS.

## Carillon Device Design and Implantation

The Carillon Mitral Contour System (Cardiac Dimensions, Kirkland, WA) consists of a nitinol band with self-expanding anchors on either end and is delivered *via* a 9-Fr sheath in the right internal jugular vein. The device is sized on the basis of both CS diameter and CS length as defined by venography. While pre-procedural CT planning may provide some guidance in this regard, as well as an understanding of the relationship between the CS and LCx, procedural imaging is the currently established standard.

**Figure 1:**
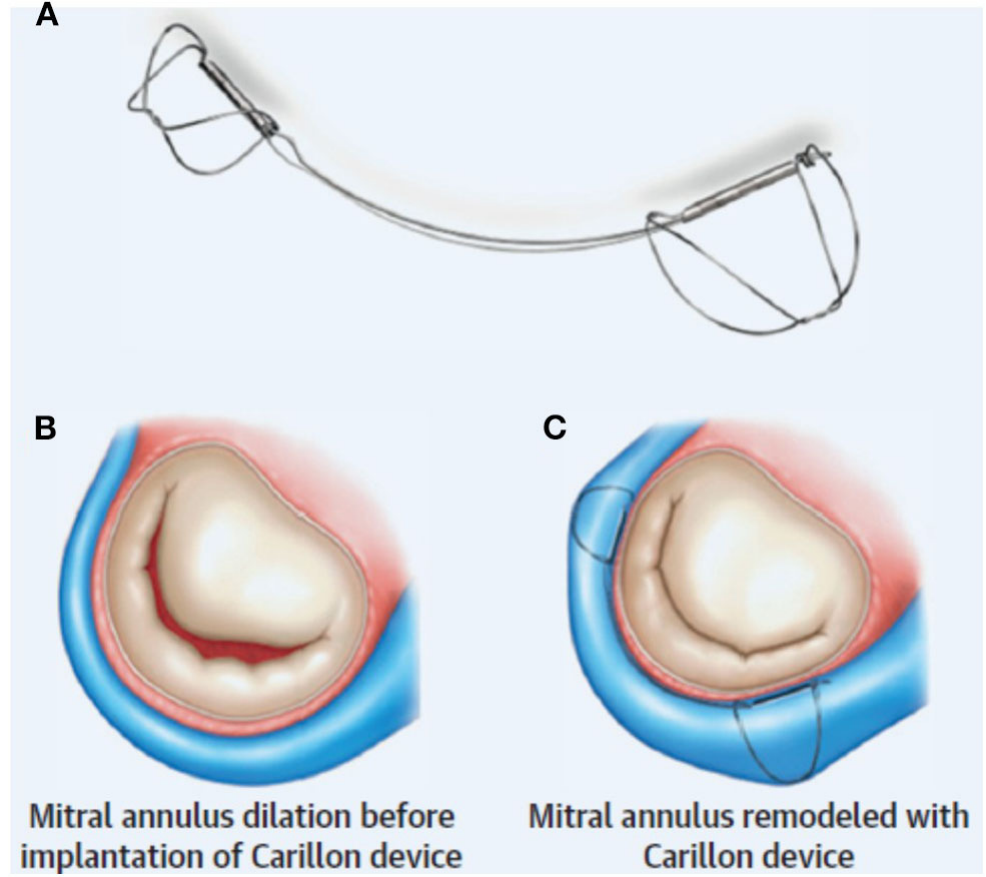
Indirect mitral annuloplasty using the Carillon device: **(A)** The Carillon consists of a band with two anchors. **(B)** Annular dilation is **(C)** improved with placement of the device resulting in improved MV coaptation. Reproduced from Witte et al. (10).

Procedurally, the distal anchor is placed as far toward the lateral annulus as possible (just proximal to the anterior interventricular vein) and then tension is applied in order to “cinch” the annulus (Figure 1). The degree of tension necessary is decided on the basis of reduction in MR (with procedural echocardiography). Once this step is completed, the proximal anchor is deployed, and coronary angiography is performed to evaluate for LCx compression (discussed below). If necessary, the Carillon can be retrieved at any point until the final release from the delivery system.

## Clinical Evidence Base

Indirect CS annuloplasty using the Carillon device has taken a deliberate pathway from case series to randomized trials, allowing iterative improvements in device design and construction as operators improve their shared experience.

### Early Trials of the Carillon

The AMADEUS (CARILLON Mitral Annuloplasty Device European Union Study), TITAN (Tighten the Annulus Now), and TITAN II trials were conducted to establish the safety and efficacy of the Carillon. Clinical feasibility was first evaluated in AMADEUS using the CARILLON XE device (11). Among 48 patients with FMR and LV systolic dysfunction, the device was successfully implanted in 30 patients. There was a significant decrease in mitral annulus diameter [from 4.2 to 3.78 cm (10%)], MR (average reduction, 23%), and New York Heart Association (NYHA) class (from 2.9 to 1.8), as well as improvement in the quality-of-life score and 6-minute walk testing (from 307 to 403 meters) at 6-month follow-up. Eighteen patients did not receive the device due to: CS-related complications (*n* = 3), fluoroscopic equipment failure (*n* = 2), or retrieval of the device after implantation because of inadequate MR reduction or coronary compromise (*n* = 13). Six patients (13%) experienced a total of seven complications within 30 days of the procedure: one patient died of multiorgan failure, three experienced myocardial infarction (none requiring percutaneous coronary intervention), and three experienced CS dissection or perforation. The complications were clustered early in the experience and improvement in safety was observed later in the study on the basis of changes to the implantation procedure. On the basis of this early work, the CARILLON system was granted the CE mark of approval for use in Europe.

The TITAN study enrolled 53 patients after some device improvements to that used in AMADEUS, and comparison was made between the 36 patients with successful implantation vs. the 17 in whom the device was retrieved. Patients were relatively young (mean age, 62 years) with a mean left ventricular ejection fraction (LVEF) of 28% and NYHA class III symptoms, and mortality was similar in both groups (1.9% at 30 days and 22.6% at 12 months). Those patients with successful device implantation (68%) experienced an average 50% reduction in regurgitant volume in comparison to stable volume in non-implanted patients, along with significant reduction in LV systolic and diastolic volumes were significantly compared with an increase in volumes in the non-implanted group. Reduction in NYHA class from III to II was sustained in the implanted group out to 2 years (12).

The TITAN II trial enrolled 36 patients with average age 70 years and average LVEF 34% for treatment with the newer generation CARILLON mXE2 device, which had undergone modifications due to device fractures that occurred during TITAN. Of these, 30 had successful implantation (83%), and the other six had uncomplicated device retrieval due to coronary compromise. The one implanted patient who died (2.8%) within 30 days died on post-procedure day 17 during treatment for acute cholecytitis. Results were effective and durable, with 1-year improvement in 6MWT from 284 to 381 m and 75% of patients had 2+ MR or less. There was no change in the 15% reduction achieved in mitral annular dimension seen acutely over the next year.

### Randomized Trials of the Carillon

The most recent data regarding the CARILLON system was provided by the randomized, double-blind REDUCE-FMR (Carillon Mitral Contour System for Reducing Functional Mitral Regurgitation) trial (10). One-hundred and 20 patients with an average age of 70 years and LVEF 34% were randomized in a 3:1 fashion to device (*n* = 87) vs. medical management alone (*n* = 33). Importantly, of the device group, only 12 patients (14%) could not be implanted due to anatomic issues (eight with LCx impingement, two with CS dissection, and two with failure of the distal anchor to maintain tension). The current contemporary iteration of the device therefore demonstrates a greater degree of procedural success/implantation than the earlier trials.

**Figure 2:**
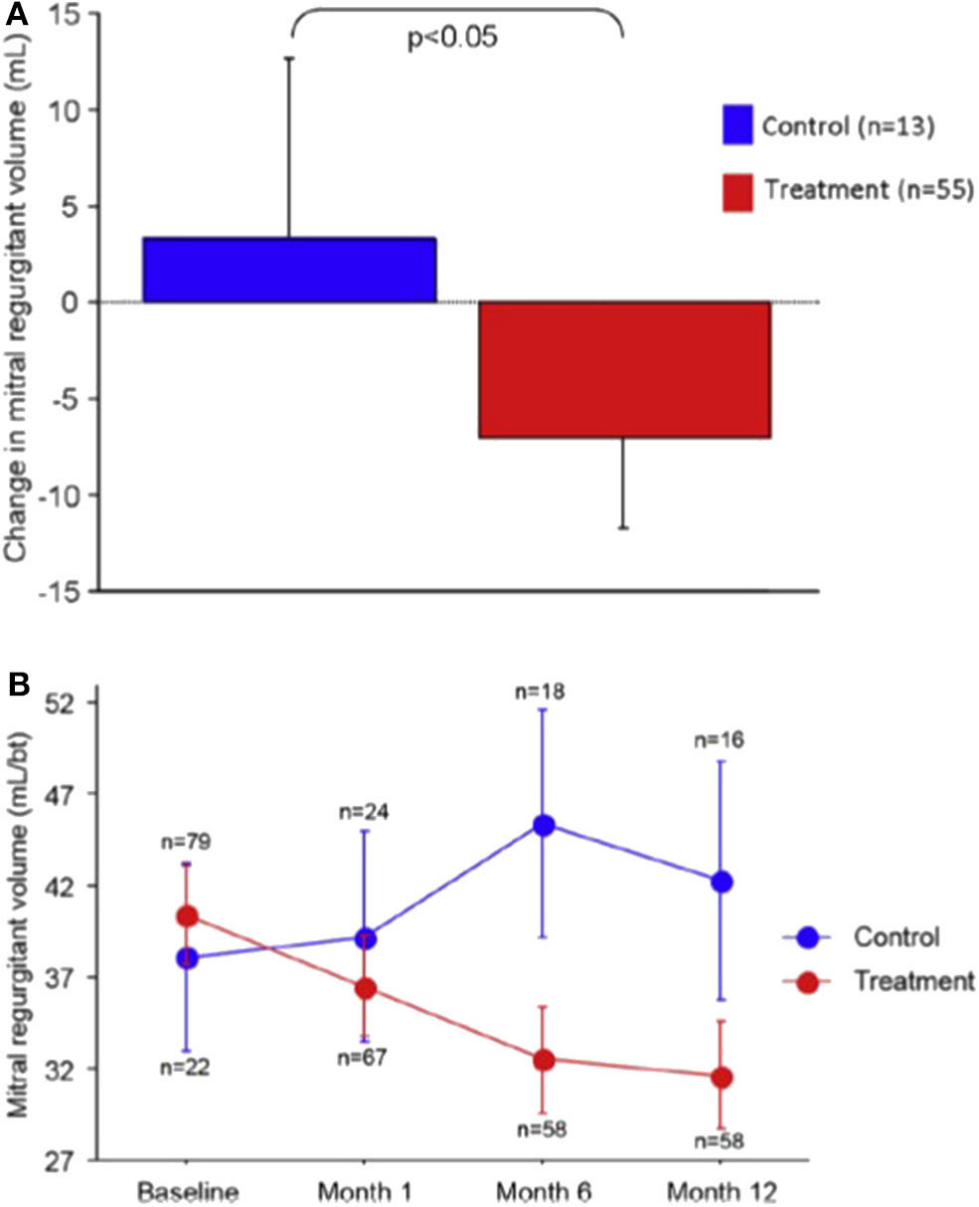
Reduction in MR volume in the REDUCE-FMR trial: **(A)** Mean change in regurgitant volume at 12 months and **(B)** Reduction various time-points over the first year. Reproduced from Witte et al. (10).

At 12 months, the primary endpoint of regurgitant volume (Rvol) reduction was significantly better in the treatment group (Rvol reduction 7.1 ml/beat vs. increase 3.3 ml/beat, *p* = 0.049) (Figure 2). There was also significant improvement in LV remodeling as demonstrated by a LV diastolic volume decrease of 10.4 ml vs. an increase of 6.5 ml (*p* = 0.03) and LV systolic volume decrease of 6.2 ml vs. an increase of 6.1 ml (*p* = 0.04). Secondary functional outcome measures demonstrated statistically significant improvements in NYHA class and 6-min walk testing. Importantly, there was no procedural perforations or device fracture or embolization. Three patients in the device group suffered a myocardial infarction within 30 days, one of which was due to LCx compression. Two patients in whom a device had been implanted died within 30-days of progressive heart failure.

The CARILLON trial (NCT03142152) has begun enrollment though is currently on hold due to the COVID-19 pandemic. This will be largest trial of the device with an intent to enroll 400 patients at 50 centers in the US and Europe. Patients will be randomized in a 1:1 fashion to device vs. a sham procedure (both in addition to guideline-directed medical therapy). There are multiple unique features to this trial. First, patients can be enrolled with 2+ FMR, which is beneficial both due to the variable severity of regurgitation in patients with FMR as well as the potential for improving long-term clinical and echocardiographic parameters by treating the disease earlier in its course. Second, patients who remain symptomatic and with severe MR (in either the treatment or sham arm) can have advanced treatment at any time (as determined by the eligibility committee) to include surgery, advanced heart failure mechanical support, or MitraClip. In this regard, the trial will be a demonstration of “real-world” practice with the possibility to better understand sequential annular and edge-to-edge repair.

## Durability and Cardiac Remodeling

Based on the limited number of patients treated and data available from clinical trials, it is difficult to comment accurately on the durability of the Carillon indirect annuloplasty. While the REDUCE FMR trial showed that, at 12-months, approximately one-quarter of treated patients had worsening MR, it is not clear what proportion (if any) of these patients had an initial improvement post-procedure (as opposed to a surgical group of MVRe where all patients start out with no/minimal MR immediately after surgery). However, the evidence provided of reduction in LV volumes and an overall reduction in regurgitant volume in time-points over the first 12 months is encouraging.

Recently, Ruf et al. (13) provided their analysis of 37 patients who had undergone Carillon implantation with comparison of atrial and ventricular dimensions at baseline, 30-days, and 1 year. The found that among the treated patients, there was a significant early reduction in annular dimensions (~15% in diameter) as well as in LA volume (23%) despite an insignificant reduction in LV end diastolic volume. Further, these changes were sustained at 1 year.

A direct comparison to surgical mitral annuloplasty cannot be made on the basis of patient-level data. However, an interesting observation is that among patients who have undergone surgical annuloplasty, a larger MV tenting area is a predictor of MR recurrence (14). On the other hand, a recent analysis of data from the three major Carillon trials (TITAN, TITAN II, and REDUCE-FMR) found that patients who had a higher degree of tenting actually did better than their counterparts with less MV tenting (15). It is possible the design of the indirect annuloplasty allows the annulus to retain its native annular function (and thereby its contribution to ventricular function), though this is of course purely conjectural.

Taken together, the positive remodeling of LA and LV provided by the Carillon provides encouragement (among those patients who respond well to the therapy) with regard to long-term benefits with regard to SMR reduction. Further, maintenance of annular motion may also contribute to sustained long-term outcomes in these patients.

## Patient Selection

Each of the percutaneous mitral repair devices has been thus far used to a limited degree, and cross-device trial data is lacking. However, with regard to the Carillon itself, an attractive feature is that it does not interact at all with the MV itself (for instance, as does traditional edge-to-edge repair). Therefore, it can be seen as a “first step” for patients who are anatomically feasible for treatment, followed by reassessment if necessary for additional therapies such as a subsequent MitraClip or NeoChord. It may even present as an especially attractive strategy for those patients who cannot be effectively treated by MitraClip alone due to significant coaptation gap or regurgitation all along the coaptation line; approximation of the MV leaflets even to some degree may allow operators to provide a better clip repair.

Given the anatomic relationship of the atrial tissue with the mitral annulus, it is possible that the Carillon could be uniquely suited to patients with “atrial FMR” by providing a reduction in atrial and mitral annular dimension. However, as the current clinical trials have specifically targeted patients with LV dysfunction and SMR, at this time such a statement is only hypothetical.

## Potential Complications

Use of the Carillon in clinical trials and commercial experience represents the most robust mitral repair device outside of the MitraClip. Importantly, the procedure is relatively straight-forward in comparison to these other devices, with average device implant time just over 1 h in the REDUCE-FMR trial, which included mostly new operators. Additionally, as a primarily right-sided/venous procedure, safety is quite high with a relatively low rate of significant complications related to the device. In addition to typical complications of catheter-based procedures such as access-site trauma and cardiac chamber perforation, some specific issues related to Carillon implantation should be considered.

### Dissection of the CS

As a venous structure, the CS is thin walled. Dissection may be due to tortuous anatomy, venous valves, forceful catheter/device manipulation, or injection into the wall of the vessel. In a recent analysis of 5,011 patients undergoing CS lead placement for cardiac resynchronization therapy (CRT), 35 (0.7%) experienced CS dissection (six with perforation) (16). Among the 29 patients with dissection alone, 21 (72%) had a CS lead still placed successfully, usually by either finding the true lumen or selecting a more proximal location/branch in which to place the lead. Among the remaining patients, six underwent successful CS lead placement between 9 days and 10 months. Taken together, these data are consistent with earlier reports demonstrating resolution in venous patency of the CS on follow-up venography within 2–3 months (17).

**Figure 3:**
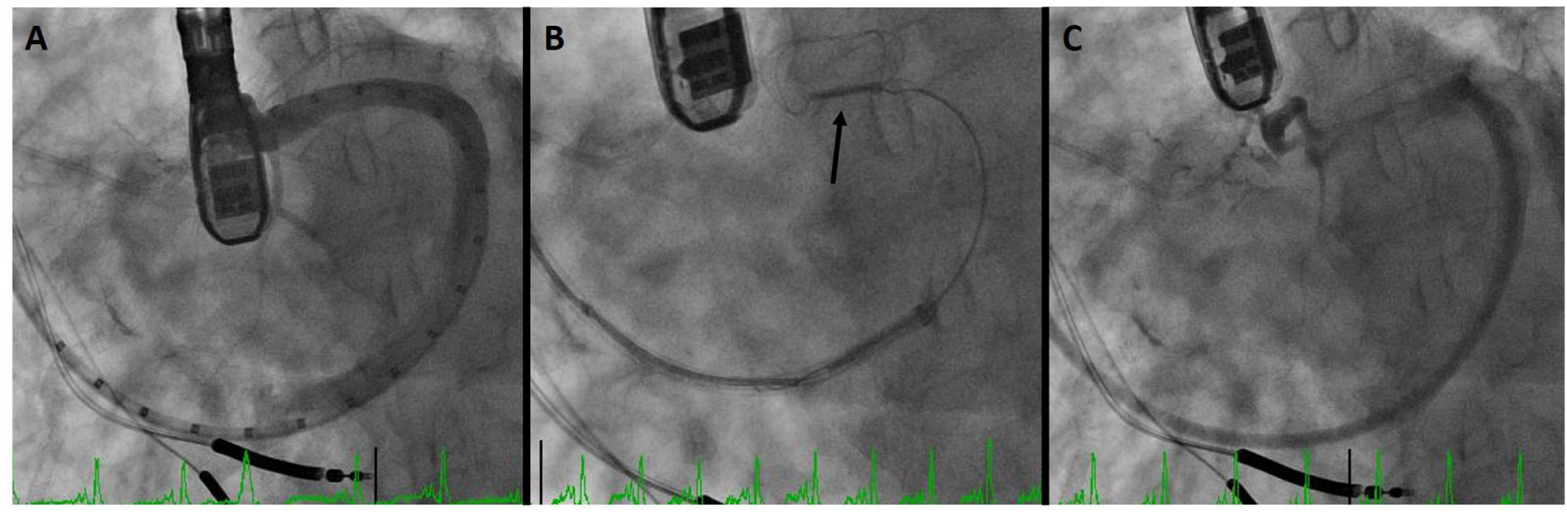
Coronary sinus (CS) dissection: **(A)** sinus venography for device sizing. **(B)** Deployment of the distal anchor (arrow) and cinching of the annulus. **(C)** Venography after device recapture due to circumflex coronary compression demonstrates dissection of the CS.

Figure 3 demonstrates a CS dissection in a patient who presented for Carillon implantation. Due to impingement of the circumflex coronary artery by the distal anchor after tension was applied, the device was recaptured with the intent to deploy it more proximally. However, venography to reassess the CS and identify a suitable location demonstrated dissection of the vessel. Anecdotally some operators have mentioned placement of a device despite CS dissection, though in this patient the procedure was aborted in order to provide time for healing of the vascular trauma.

### Circumflex Coronary Impingement

**Figure 4:**
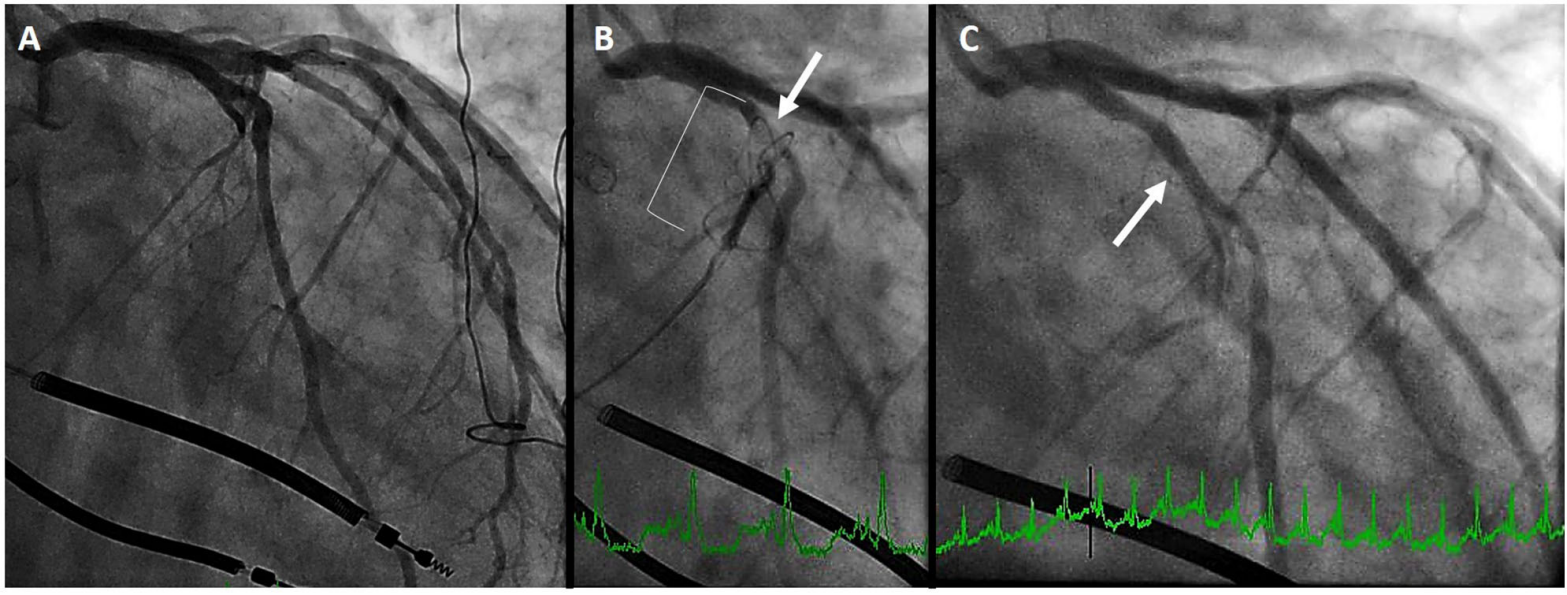
Left circumflex coronary artery compression. **(A)** initial coronary angiogram. **(B)** distal anchor (bracket) causing compression of the proximal circumflex (arrow) **(C)** angiography after recapture of the Carillon demonstrates resolution of circumflex compression (arrow).

In the aforementioned analysis of cardiac CT, Choure et al. demonstrated that the LCx coronary artery crosses between the CS and MVA in 80% of patients. While the location of this crossing is variable, allowing CS annuloplasty in a great number of patients, it is important to be cognizant of this complication. Among the three initial trials of the Carillon previously discussed, coronary compression precluded device placement in 10–15% of patients. In REDUCE-FMR, eight patients of the 87 (9%) planned for a device could not receive one due to coronary compression (which resolved in all with device recapture). Figure 4 demonstrates a patient in whom the proximal circumflex was compressed by the distal anchor. As is common, the compression was relieved by recapture of the device.

## Conclusions

There are a number of interesting strategies and devices aimed at treating patients with functional mitral valve regurgitation. Indirect mitral annuloplasty utilizing the CS is an important option and is commercially available in Europe. Early data using the Carillon device has demonstrated promise with regard to improvements in regurgitant volume, LV remodeling, and functional patient outcomes. The large, randomized Carillon trial that is currently ongoing will provide additional important outcomes data and is eagerly anticipated.

## Author Contributions

AK and SK were involved significantly in creating this manuscript. All authors contributed to the article and approved the submitted version.

## Conflict of Interest

SK is one of the principal investigators of the Carillon Trial but does not receive any financial compensation for his role. The remaining author declares that the research was conducted in the absence of any commercial or financial relationships that could be construed as a potential conflict of interest.
